# Pneumococcal colonization in pediatric patients undergoing hematopoietic cell transplantation

**DOI:** 10.1128/spectrum.04006-25

**Published:** 2026-06-01

**Authors:** Liset Olarte, Jennifer E. Schuster, Ibrahim Ahmed, Kristina G. Hulten

**Affiliations:** 1 Division of Infectious Diseases, Department of Pediatrics, Baylor College of Medicine 3989, Houston, Texas, USA; 2 Division of Infectious Diseases, Department of Pediatrics, Children’s Mercy-Kansas City 4204 , Kansas City, Missouri, USA; 3 Division of Hematology/Oncology/Bone Marrow Transplant, Department of Pediatrics, Children’s Mercy-Kansas City 4204 , Kansas City, Missouri, USA; bioMerieux Inc., Salt Lake City, Utah, USA

**Keywords:** *Streptococcus pneumoniae*, colonization, hematopoietic cell transplant

## Abstract

Despite the increased risk of invasive pneumococcal disease among pediatric hematopoietic cell transplant (HCT) recipients, pneumococcal colonization has not been evaluated in this population. We prospectively assessed longitudinal pneumococcal colonization in 22 pediatric HCT recipients from conditioning therapy to 100 days post-HCT. A higher likelihood of pneumococcal colonization was observed in the late post-HCT period (weeks 9–14) compared with the early post-HCT period (weeks 1–8), with odds increasing with each additional week after conditioning.

**IMPORTANCE:**

This is the first study to longitudinally evaluate pneumococcal colonization in pediatric hematopoietic cell transplant (HCT) recipients from the onset of conditioning therapy to 100 days post-HCT. We found a higher likelihood of pneumococcal colonization in the late post-HCT period (weeks 9–14) compared with the early post-HCT period (weeks 1–8), with odds increasing with each additional week after conditioning. Despite study limitations, these findings provide foundational insight into early post-HCT colonization dynamics and highlight the need for larger studies to better understand the roles of antibiotic exposure and pre-HCT vaccination in reducing pneumococcal colonization.

## OBSERVATION

Nasopharyngeal colonization by *Streptococcus pneumoniae* is an essential step for the development of invasive pneumococcal disease (IPD). Hematopoietic cell transplant (HCT) recipients are at a significantly greater risk of IPD than other immunocompromised patients (1). A systematic review reported a pooled IPD incidence of 696 and 812 per 100,000 person-years in autologous and allogeneic HCT recipients, respectively, compared to an incidence of 465 per 10,000 person-years in solid-organ transplant (SOT) recipients (2). Moreover, HCT recipients develop IPD almost a year earlier than SOT recipients (3). This is thought to be secondary to the preceding immunocompromised status of HCT recipients and the high levels of immunosuppression therapy required for HCT. Furthermore, HCT recipients who develop chronic graft-versus-host disease are at a substantially higher risk of IPD than other HCT recipients; thus, penicillin prophylaxis is recommended for those patients (4).

Despite the increased risk of IPD among HCT recipients, pneumococcal colonization in pediatric HCT recipients has not been evaluated. In the United States, HCT recipients receive antibacterial prophylaxis, particularly fluoroquinolones, at least until engraftment, and broad-spectrum beta-lactam antibiotics when febrile, both of which may affect pneumococcal colonization (5). Understanding pneumococcal colonization in HCT recipients at different time points after HCT is critical for the implementation of pneumococcal re-immunization strategies and other preventive measures. Our objective was to characterize the pneumococcal colonization rates among pediatric HCT recipients from onset of conditioning therapy pre-HCT to 100 days post-HCT, a critical period of profound immunosuppression, mucosal barrier disruption, and high susceptibility to infections.

Mid-turbinate samples (MTS) from pediatric patients undergoing HCT at Children’s Mercy Kansas City (CMKC) from September 2015 to January 2017 and who were prospectively enrolled in a longitudinal surveillance study (6) evaluating the weekly prevalence of respiratory viruses in HCT recipients were included in this study.

Study population and specimen collection have been reported previously (6). Briefly, MTS were obtained during conditioning therapy (week −1), during HCT (week 0), and weekly ±3 days for the first 100 days after HCT (weeks 1–14) using nylon flocked swabs (FLOQSwabs) and were placed in Copan Universal Transport Medium. An aliquot of each MTS was shipped to the Infectious Diseases Research Laboratory at Texas Children’s Hospital, Houston, TX, for pneumococcal detection. Bacterial DNA was extracted from MTS using the QIAGEN DNeasy Automated Extraction System. For *S. pneumoniae* detection, 5 μL of extracted DNA were used for real-time PCR using the Centers for Disease Control and Prevention’s *lytA* primer (autolysin A-encoding gene) (7). A cycle threshold value ≤35 was considered positive.

Demographic and clinical information was obtained from the longitudinal surveillance study database, and pneumococcal immunization status was obtained from electronic medical records. During the study period, CMKC guidelines for antibacterial prophylaxis and fever management in HCT recipients recommended that all patients undergoing high-dose chemotherapy followed by HCT receive antibacterial prophylaxis with an antipseudomonal fluoroquinolone (preferably ciprofloxacin) starting on day −1 of conditioning therapy, unless contraindicated. Prophylaxis was continued until neutrophil engraftment (absolute neutrophilic count >500/mm^3^) or the development of fever. Empiric treatment of fever consisted of cefepime, with vancomycin added based on clinical status. Use of ciprofloxacin prophylaxis or empiric or definitive *S. pneumoniae*-active antibiotic therapy (SPAT) was recorded during the weeks MTS were collected. Pneumococcal revaccination at CMKC typically begins 4 months post-HCT.

The post-HCT period was divided into two intervals (weeks 1–8 and weeks 9–14) to evaluate temporal trends in pneumococcal colonization. Weeks 1–8 represent the peri-engraftment period, during which patients are typically neutropenic, profoundly immunosuppressed, and often maintained on broad-spectrum antimicrobials due to their high risk for infections. In contrast, weeks 9–14 reflect a period during which prophylactic antimicrobial regimens may be de-escalated, and exposure to external environments may increase.

Patient characteristics were reported as frequencies and proportions for categorical variables and as median and interquartile range (IQR) for continuous variables. Differences between patients with and without pneumococcal colonization were assessed using the Wilcoxon rank-sum test for continuous variables and Fisher’s exact test for categorical variables. Longitudinal generalized estimating equations (GEE) modeling, adjusted for age at enrollment, SPAT, and type of transplant, was used to assess the odds of pneumococcal colonization from conditioning therapy through week 14 post-HCT using follow-up time in weeks and period-based analysis (difference between HCT periods). Because no pneumococcal colonization events occurred among vaccinated participants, vaccine status resulted in complete separation and was omitted from GEE models. Differences in viral detection between HCT periods were also evaluated. Analyses were performed on Stata version 19.5 (StataCorp LLC, College Station, TX, USA). A *P* value of <0.05 was considered statistically significant.

**Fig 1:**
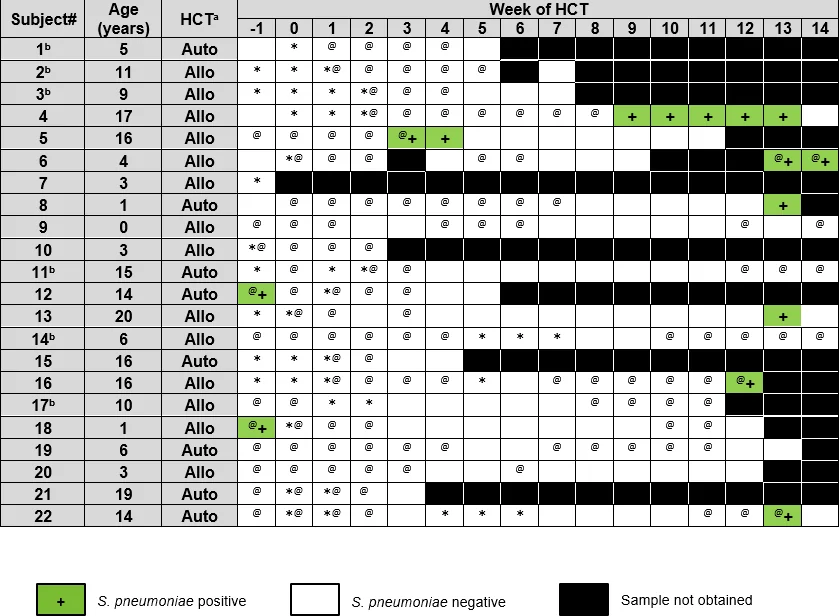
*Streptococcus pneumoniae* detection from the onset of conditioning therapy to 100 days post-hematopoietic cell transplantation. ^a^Type of HCT. Allo, allogeneic; auto, autologous. ^b^Documented pneumococcal conjugate vaccine administration prior to conditioning regimen. *Ciprofloxacin prophylaxis. ^@^*S. pneumoniae*-active antibiotic therapy. In order of frequency, antibiotics used include cefepime, vancomycin, meropenem, piperacillin/tazobactam, ceftriaxone, levofloxacin, clindamycin, azithromycin, ampicillin/sulbactam, linezolid, amoxicillin/clavulanate, trimethoprim/sulfamethoxazole, and daptomycin.

Twenty-two HCT recipients were included in this study, and 253 MTS were obtained. Samples were obtained during the conditioning regimen (week −1) in all patients. Weekly samples were obtained during weeks 1–8 post-HCT in 95.5% (21/22) of patients and during weeks 9–14 post-HCT in 63.6% (14/22) of patients (Fig. 1). Patient characteristics are presented in Table 1. Six patients had documentation of receiving ≥1 of 7-valent and/or 13-valent pneumococcal conjugate vaccine prior to conditioning regimen; this information was not available for the other patients.

**TABLE 1:** Study population characteristics by pneumococcal colonization status^*a*^

	Total (*N* = 22)	Not colonized (*N* = 13)	Colonized (*N* = 9)	*P*-value
Age (years), median (IQR)	9.5 (3.0, 16.0)	6.0 (3.0, 11.0)	14.0 (4.0, 16.0)	0.30
Sex				0.81
Male	14 (63.6)	8 (61.5)	6 (66.7)	
Female	8 (36.4)	5 (38.5)	3 (33.3)	
Underlying disease				0.34
Oncologic	15 (68.2)	9 (69.2)	6 (66.7)	
Hematologic	5 (22.7)	2 (15.4)	3 (33.3)	
Immunodeficiency	2 (9.1)	2 (15.4)	0 (0.0)	
Type of transplant				0.81
Autologous	8 (36.4)	5 (38.5)	3 (33.3)	
Allogeneic	14 (63.6)	8 (61.5)	6 (66.7)	
Pneumococcal vaccine (≥1 dose)				**0.02^*b*^**
Unknown	16 (72.7)	7 (53.8)	9 (100.0)	
Yes	6 (27.3)	6 (46.2)	0 (0.0)	

^*a*^ Values are in *n* (%) unless otherwise specified. IQR, interquartile range.

^*b*^ Boldface value represents a statistically significant result.

During weeks 1–8 post-HCT, all 21 patients with available data received either ciprofloxacin prophylaxis or SPAT. One patient received ciprofloxacin prophylaxis for 2 weeks and no further antibiotics until week 8. Nine patients initially received ciprofloxacin prophylaxis and were later switched to SPAT. The remaining 11 patients received SPAT, which continued for 1–7 weeks. In contrast, during weeks 9–14, no patients received ciprofloxacin prophylaxis. Of the 14 patients evaluated during this period, 9 (64.3%) received SPAT for a duration of 2–5 weeks.

Overall, 40.9% (9/22) of patients were colonized with *S. pneumoniae* (Table 1). Pneumococcal colonization occurred in 9 of 16 patients with unknown PCV vaccination history, while none of the 6 patients with confirmed pre-HCT PCV vaccination were colonized. In longitudinal GEE analyses, the odds of pneumococcal colonization increased over time following HCT (Supplemental material). When follow-up time was modeled continuously, each additional week post-conditioning was associated with higher odds of pneumococcal colonization (OR 1.27, 95% CI 1.03–1.57; *P* = 0.03). In period-based analyses, pneumococcal colonization was significantly more frequent during the late post-HCT period (weeks 9–14) compared with the early post-HCT period (weeks 1–8) (OR 15.59, 95% CI 1.56–155.97; *P* = 0.02), whereas colonization during conditioning/HCT (week −1 to 0) did not differ significantly. Age at enrollment, SPAT, and transplant type were not independently associated with pneumococcal colonization in either model.

In contrast, no significant differences in viral detection were observed between early and late post-HCT periods (OR 0.97, 95% CI 0.32–2.90; *P* = 0.95). Co-detection of *S. pneumoniae* and a respiratory virus was rare. Only subject #8 on week 13 post-HCT tested positive for *S. pneumoniae* and rhinovirus C.

During the study period, only one of the HCT recipients developed IPD. Subject #11 was diagnosed with pneumococcal bacteremia during week 2, despite no prior detection of pneumococcal colonization. No subjects received post-HCT pneumococcal immunization during the study period.

Pneumococcal colonization has been studied in children with cancer and among adult SOT recipients (8, 9). However, to our knowledge, this is the first study to longitudinally evaluate pneumococcal colonization from conditioning therapy to the first 100 days after HCT in pediatric HCT recipients.

Our data showed a higher likelihood of pneumococcal colonization in the late post-HCT period (weeks 9–14) compared with the early post-HCT period (weeks 1–8), with odds increasing with each additional week after conditioning. Interestingly, pneumococcal colonization was observed even during weeks in which SPAT was administered; accordingly, SPAT use was not significantly associated with pneumococcal colonization in the GEE model. We included antibiotic use as a potential confounding factor. Therefore, the analysis was not intended to assess the effectiveness of prophylactic or empiric antibiotics in preventing colonization. One hypothesis is that the rise in colonization during weeks 9–14 may reflect early nasopharyngeal microbiota recovery as antibiotic pressure decreases and patients gradually resume exposure to community and household environments. Prior studies have shown that antibiotic exposure can suppress pneumococcal colonization (8). For example, Italian data in children with cancer reported that trimethoprim-sulfamethoxazole prophylaxis was significantly associated with reduced pneumococcal colonization (8).

Although some patients in our study had received pneumococcal vaccines prior to HCT, we were unable to assess the impact of pre-HCT immunization on post-HCT colonization due to incomplete vaccination records. However, none of the patients with documented pre-HCT PCV receipt were colonized, whereas 56% of those with unknown vaccination history were colonized. Although biologically plausible, this observation was limited by small numbers and potential confounding from antibiotic exposure and should be interpreted with caution. Regardless, pneumococcal re-vaccination starting at 3–6 months after HCT remains an essential preventive strategy post-transplant (10).

Our study has several limitations. First, the small sample size and single-center design limit the generalizability of our results. Second, although we recorded weekly antibiotic exposure, we lacked granular data on the precise timing and duration of antibiotic use relative to MTS collection, which limits our ability to analyze dose-response relationships between antibiotic use and pneumococcal colonization. Third, we did not collect data on household or community exposures, which are known risk factors for pneumococcal colonization, particularly contact with young children. Finally, we did not perform pneumococcal serotyping or assess antimicrobial resistance, both of which would have provided important information on vaccine coverage and resistance patterns.

Despite these limitations, our study provides valuable initial longitudinal data on the dynamics of pneumococcal colonization in pediatric HCT recipients. Larger studies are needed to further characterize pneumococcal colonization dynamics and to understand the roles of antibiotic exposure and pre-HCT vaccination in the subsequent risk of IPD in HCT recipients.

## Data Availability

The data sets generated and/or analyzed during the current study are not publicly available but are available from the corresponding author on reasonable request.

## SUPPLEMENTAL MATERIAL

Supplemental material spectrum.04006-25-s0001.docx Table S1; Fig. S1.

ASM does not own the copyrights to Supplemental Material that may be linked to, or accessed through, an article. The authors have granted ASM a non-exclusive, world-wide license to publish the Supplemental Material files. Please contact the corresponding author directly for reuse.
