# Supplementary material for: Pneumococcal colonization in pediatric patients undergoing hematopoietic cell transplantation
Source: Microbiol Spectr. 2026 Jun 1;14(7):e04006-25. doi: 10.1128/spectrum.04006-25 (PMC13340042; doi:10.1128/spectrum.04006-25)
Supplement: Supplemental material — Table S1; Fig. S1. [file spectrum.04006-25-s0001.docx]

**SUPPLEMENTAL MATERIAL
Table. Longitudinal GEE analysis of pneumococcal colonization from conditioning therapy through week 14 post‑HCT**

|  | **Multivariable** | |
| --- | --- | --- |
|  | **OR (95% CI)** | **p-value** |
| **1. Use continuous follow-up time (in weeks)** |  |  |
| Follow-up time (in weeks) | 1.27 (1.03, 1.57) | 0.03 |
| Age at enrollment (years) | 1.09 (0.99, 1.20) | 0.07 |
| SPAT |  |  |
| No | (reference) |  |
| Yes | 1.42 (0.34, 5.96) | 0.63 |
| Type of transplant |  |  |
| Autologous | (reference) |  |
| Allogeneic | 2.22 (0.52, 9.43) | 0.28 |
| **2. Use categorical follow-up time (period-based)** |  |  |
| Follow-up time (period-based) |  |  |
| Conditioning / HCT (week -1 to 0) | 3.63 (0.29, 45.82) | 0.32 |
| Early post-HCT (weeks 1–8) | (reference) |  |
| Late post-HCT (weeks 9–14) | 15.59 (1.56, 155.97) | 0.02 |
| Age at enrollment (years) | 1.09 (0.99, 1.21) | 0.09 |
| SPAT |  |  |
| No | (reference) |  |
| Yes | 1.46 (0.43, 4.93) | 0.54 |
| Type of transplant |  |  |
| Autologous | (reference) |  |
| Allogeneic | 2.20 (0.52, 9.40) | 0.29 |

OR, odds ratio; CI, confidence interval; SPAT, *S. pneumoniae*-active antibiotic therapy; HCT, hematopoietic cell transplantation

**Figure. Cubic spline plot of predicted probability of pneumococcal colonization from conditioning therapy through week 14 post‑HCT**
